# Early childhood deprivation is associated with alterations in adult brain structure despite subsequent environmental enrichment

**DOI:** 10.1073/pnas.1911264116

**Published:** 2020-01-06

**Authors:** Nuria K. Mackes, Dennis Golm, Sagari Sarkar, Robert Kumsta, Michael Rutter, Graeme Fairchild, Mitul A. Mehta, Edmund J. S. Sonuga-Barke, on behalf of the ERA Young Adult Follow-up team

**Affiliations:** ^a^Department of Child and Adolescent Psychiatry, Institute of Psychiatry, Psychology and Neuroscience, King’s College London, London SE5 8AF, United Kingdom;; ^b^Department of Neuroimaging, Institute of Psychiatry, Psychology and Neuroscience, King’s College London, London SE5 8AF, United Kingdom;; ^c^Centre for Innovation in Mental Health, School of Psychology, University of Southampton, Southampton SO17 1BJ, United Kingdom;; ^d^Cognitive Neuroscience & Neuropsychiatry, Great Ormond Street Institute of Child Health, University College London, London WC1E 6BT, United Kingdom;; ^e^Genetic Psychology, Department of Psychology, Ruhr University Bochum, Bochum 44801, Germany;; ^f^Social, Genetic & Developmental Psychiatry Centre, Institute of Psychiatry, Psychology and Neuroscience, King’s College London, London SE5 8AF, United Kingdom;; ^g^Department of Psychology, University of Bath, Bath BA2 7AY, United Kingdom;; ^h^Department of Child & Adolescent Psychiatry, Aarhus University, Aarhus DK-8200, Denmark

**Keywords:** early adversity, institutional deprivation, brain structure, structural MRI, ADHD

## Abstract

Early childhood deprivation is associated with higher rates of neurodevelopmental and mental disorders in adulthood. The impact of childhood deprivation on the adult brain and the extent to which structural changes underpin these effects are currently unknown. To investigate these questions, we utilized MRI data collected from young adults who were exposed to severe deprivation in early childhood in the Romanian orphanages of the Ceaușescu era and then, subsequently adopted by UK families; 67 Romanian adoptees (with between 3 and 41 mo of deprivation) were compared with 21 nondeprived UK adoptees. Romanian adoptees had substantially smaller total brain volumes (TBVs) than nondeprived adoptees (8.6% reduction), and TBV was strongly negatively associated with deprivation duration. This effect persisted after covarying for potential environmental and genetic confounds. In whole-brain analyses, deprived adoptees showed lower right inferior frontal surface area and volume but greater right inferior temporal lobe thickness, surface area, and volume than the nondeprived adoptees. Right medial prefrontal volume and surface area were positively associated with deprivation duration. No deprivation-related effects were observed in limbic regions. Global reductions in TBV statistically mediated the observed relationship between institutionalization and both lower intelligence quotient (IQ) and higher levels of attention deficit/hyperactivity disorder symptoms. The deprivation-related increase in right inferior temporal volume seemed to be compensatory, as it was associated with lower levels of attention deficit/hyperactivity disorder symptoms. We provide compelling evidence that time-limited severe deprivation in the first years of life is related to alterations in adult brain structure, despite extended enrichment in adoptive homes in the intervening years.

## Significance

Millions of children worldwide live in nonfamilial institutions. We studied impact on adult brain structure of a particularly severe but time-limited form of institutional deprivation in early life experienced by children who were subsequently adopted into nurturing families. Institutional deprivation was associated with lower total brain volume in a dose-dependent way. Regionally specific effects were seen in medial prefrontal, inferior frontal, and inferior temporal areas. Deprivation-related alterations in total brain volume were associated with lower intelligence quotient and more attention deficit/hyperactivity disorder symptoms; alterations in temporal volume seemed compensatory, as they were associated with fewer attention deficit/hyperactivity disorder symptoms. We provide evidence that early childhood deprivation is related to alterations in adult brain structure, despite environmental enrichment in intervening years.

Neuroplasticity, the brain’s inherent ability to dynamically adapt and change in response to environmental influences, supports normal learning and development. It also promotes recovery of function following injury and insult (1). At the same time, it may leave the human brain vulnerable to the negative effects of adverse psychosocial experiences, such as maltreatment (2). This might be especially true during early childhood, which is characterized by rapid and dynamic changes in brain structure and function (3) that have been hypothesized to increase malleability to environmental influences (4). Animal experiments support this hypothesis and suggest that the amygdala, hippocampus, and prefrontal cortex are particularly vulnerable to the effects of early life stress (4), perhaps because of their protracted development and close links to the hypothalamus–pituitary–adrenal axis (5).

It is challenging to interpret findings from human early maltreatment studies, which cannot experimentally manipulate exposure to adversity for obvious ethical reasons. This is because design limitations restrict the ability to assign a causal role to such exposures (6). For instance, in many observational studies, maltreated individuals remain with their families—often the perpetrators—making it difficult to isolate early from later adverse exposures (7). Even in cases where children escape maltreatment by parents through adoption or fostering, effects of maltreatment are genetically confounded: environmental exposures, correlated brain alterations, and associated psychopathology may all be driven by common genetic risk factors passed from parent to child (6). In addition, the majority of findings are based on retrospective reports of maltreatment that show limited agreement with prospectively assessed maltreatment (8). Recruiting participants on the basis of retrospective reports may also lead to an oversampling of individuals with psychopathology (9, 10). This makes it difficult to isolate the effects of early adversity on the brain from the effects of later adversity or the brain-based manifestations of genetic risk or subsequent psychopathology (11).

While only studies in which deprivation is experimentally manipulated can definitively establish a causal link between adversity and outcomes, prospective longitudinal studies of adopted children exposed to deprivation for a time-limited period in early childhood within nonfamilial institutions rather than biological families offer the best opportunity to disentangle the effects of early adverse environmental exposures on brain development from such confounding factors. Inference about the causal role of exposure to adversity is strengthened further if children enter the institutions very early in life and the switch from deprived to nurturing adoptive rearing environment is abrupt, precisely timed, and not determined by underlying risk within the child but rather, by historical circumstances (6). The large-scale international adoption of the children discovered living in the brutally depriving Romanian orphanages at the time of the fall of the Ceaușescu regime represents an example of such a natural experiment.

To date, most studies of this cohort have focused on cognitive and mental health outcomes rather than brain development—concluding that extended deprivation is associated with increased rates of neurodevelopmental and mental disorders, which are often severe and persistent in nature (12, 13). In the English and Romanian Adoptees (ERA) study, adoptees entered the institutions in the first few weeks of life and then, spent between 2 wk and 43 mo living there before being adopted into families in the United Kingdom that provided mostly nurturing environments. Thus, adoption constituted a radical and sudden improvement in circumstances when compared with the appalling conditions experienced in the institutions. In the institutions, children were frequently malnourished and had minimal social contact, with insufficient caregiving and very little cognitive stimulation due to a lack of toys and confinement to cots (14). The ERA study included a comparison group of nondeprived adoptees from the host country placed before 6 mo of age to isolate the effects of deprivation from adoption per se. The sample was also stratified by duration of deprivation, thereby allowing a test of the effects of deprivation “dose” to further clarify the meaning of the link between deprivation and brain outcomes (14). Initial reports documented a devastating and pervasive initial effect of deprivation on cognitive and social development for most children. This was followed by subsequent rapid recovery up to the age of 6 y (14). Despite this, many individuals who spent an extended period (i.e., >6 mo) in the institutions subsequently displayed a distinctive and highly impairing combination of increased symptom rates of neurodevelopmental disorders, including attention deficit/hyperactivity disorder (ADHD), autism spectrum disorder (ASD), and disinhibited social engagement (DSE; a pattern of indiscriminate friendliness toward strangers and lack of selectivity in attachment-related behaviors [15]), which has persisted in many individuals through to young adulthood (12, 16). In contrast, the marked cognitive impairments seen in childhood have gradually remitted over time such that, by adulthood, most adoptees are within the normal range (12).

Here, we harness the strengths of the ERA study design to provide evidence of a specific association between exposure to deprivation limited to early childhood and altered brain structure in young adulthood. We asked if early deprivation was associated with alterations in the adult brain in terms of both global volume and regional structural metrics. The handful of studies that have examined the links between institutional deprivation and brain structure in childhood and adolescence are consistent in finding reduced total gray and white matter volumes (17–22). Results are, however, inconsistent with regard to the loci of regional effects of deprivation, perhaps because of problems with reproducibility of findings from studies using small samples combined with the different developmental stages of the assessments. There is some evidence for alterations in volumes in the prefrontal cortex (19), amygdala, and hippocampus (18–22) as well as cortical thinning in prefrontal, parietal, and temporal regions (23). Nevertheless, there have not been any systematic investigations that assess multiple morphometric measures simultaneously to test whether deprivation-related alterations in volume reflect changes in cortical thickness, surface area, or gyrification. Furthermore, none of the above studies have investigated the impact of severe deprivation on brain structure in adulthood (the mean age of the oldest sample studied to date was 16 y [18]).

In this study, we used a comprehensive whole-brain analysis strategy to first examine whether early institutional deprivation is associated with alterations in total brain volume (TBV) in young adulthood. We did this by comparing Romanian adoptees with nondeprived UK adoptees and also, by investigating associations with deprivation duration. We also tested whether any changes persisted after covarying for the potential confounders of adult body height, birth weight, and subnutrition. In an exploratory analysis, we examined the potential role of genetic confounders by testing whether polygenic scores for intracranial volume accounted for the effects of deprivation duration. Statistically taking account of these confounding factors is especially important in order to control for the possibility that later adoption (thus, extended deprivation) is linked to genetic or environmental risk (perhaps because of selection factors determining which children were adopted early vs. late) rather than deprivation exposure per se. The links between deprivation and localized changes in cortical volume, surface area, thickness, and gyrification and subcortical volumes were then explored, controlling for TBV. Based on previous studies in institutionalized children, we predicted a deprivation-related reduction in TBV and hypothesized that this effect would persist after controlling for available information on genetic and environmental confounds. Above and beyond such effects, regionally specific effects on cortical (prefrontal, parietal, and temporal lobes) and subcortical (limbic) areas were predicted. As cortical surface area is relatively less established at birth compared with cortical thickness and gyrification (24, 25), we predicted that it would be more vulnerable than the other measures to deprivation-related effects.

Our second question was if global and regional deprivation-related brain alterations statistically mediate adult neurodevelopmental and cognitive outcomes. A previous study based on the Bucharest Early Intervention Project sample reported that cortical thinning in frontal, parietal, and temporal cortices mediated the effects of institutional deprivation on inattentive symptoms in childhood (23). However, other studies have reported brain structural differences following early maltreatment in the absence of psychopathology (26). This has led researchers to propose that maltreatment-related brain alterations might in some cases represent compensatory changes, which promote resilience from psychopathology, rather than increased risk for disorders (26). This hypothesis has rarely been tested in humans (27). Given the passage of time and the wide range of postdeprivation experience since exposure, we predicted that early deprivation would be associated with adult brain structure in heterogeneous ways—some would manifest as structural markers of disorder risk (i.e., mediating poor outcomes of deprivation), and some would manifest as compensatory processes (i.e., mediating positive outcomes despite deprivation). Based on prior findings from nondeprived populations, we predicted that deprivation-related reductions in TBV would be related to low IQ (28) and higher levels of ADHD symptoms (29). Over and above this, we made the general prediction that brain regions implicated in neurodevelopmental outcomes in nondeprived samples would also be implicated in deprivation-related outcomes. For example, we hypothesized that ADHD symptoms in this sample would be linked to structural alterations within the prefrontal and temporal cortices, similar to those observed in nondeprivation-related variants of ADHD (30).

## Results

### Associations between TBV Institutional Deprivation.

**Fig. 1.**
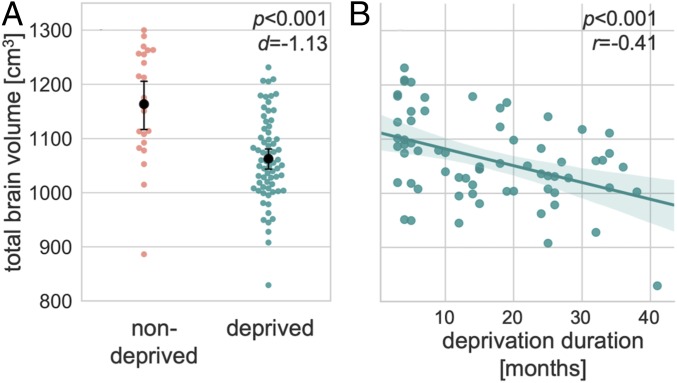
Deprivation-related differences in TBV. (*A*) Point and swarm plot depicting distributions of TBV in deprived and nondeprived groups (*n* = 88). Black whiskers show 95% CIs around the means (black dots). (*B*) Negative correlation between deprivation duration and TBV (*n* = 67). The shaded area depicts the 95% CI around the regression line. These analyses were adjusted for the effects of sex. Effect sizes were calculated with Cohen’s *d* and Pearson’s *r*.

The group of institutionally deprived Romanian adult adoptees displayed an 8.57% reduction in TBV compared with the nondeprived group of UK adoptees: *F*(1,85) = 20.55, *P* < 0.001, SE = 21.99, and Cohen’s *d* = −1.13 (Fig. 1*A*). Within the deprived group, as deprivation duration increased, TBV decreased: β = −0.31, *r*_*partial*_ = −0.41, *t* (64) = −3.62, and *P* < 0.001 (Fig. 1*B*). Each additional month of deprivation was associated with a 3.00-cm^3^ (0.27%) reduction in TBV. Results were similar for total gray and white matter volumes (*SI Appendix*, Fig. S1).

### Potential Contributing Factors.

Deprivation duration remained a significant predictor of TBV after covarying for physical height [β = −0.20, *t* (53) = −2.19, *P* = 0.03], suggesting that effects were not simply a reflection of more general deprivation-related reductions in overall growth, which were also very common in our sample (31). There was no evidence that those exposed to extended institutional deprivation had experienced more prenatal adversity [as indexed by birth weight; β = 0.12, *t* (55) = 0.90, *P* = 0.37] (*SI Appendix*, Fig. S2), and covarying for this factor did not alter the results. Subnutrition was also unlikely to account for these effects, as the relationship between children’s weight at the time that they were adopted and TBV was not statistically significant [β = 0.19, *t* (57) = 1.91, *P* = 0.06] (*SI Appendix*, Fig. S2); covarying for this factor did not change the findings. However, the composition of the participants’ diet was not measured directly. Finally, there was no association between duration of deprivation and polygenic scores for intracranial volume [β = 0.09, *t* (46) = 0.58, *P* = 0.56] (*SI Appendix*, Fig. S2), providing no evidence for the possibility that individuals with a genetic propensity toward smaller brains were adopted later, and covarying for these scores did not change the results.

### Local Alterations in Cortical Structure following Institutionalization.

**Fig. 2.**
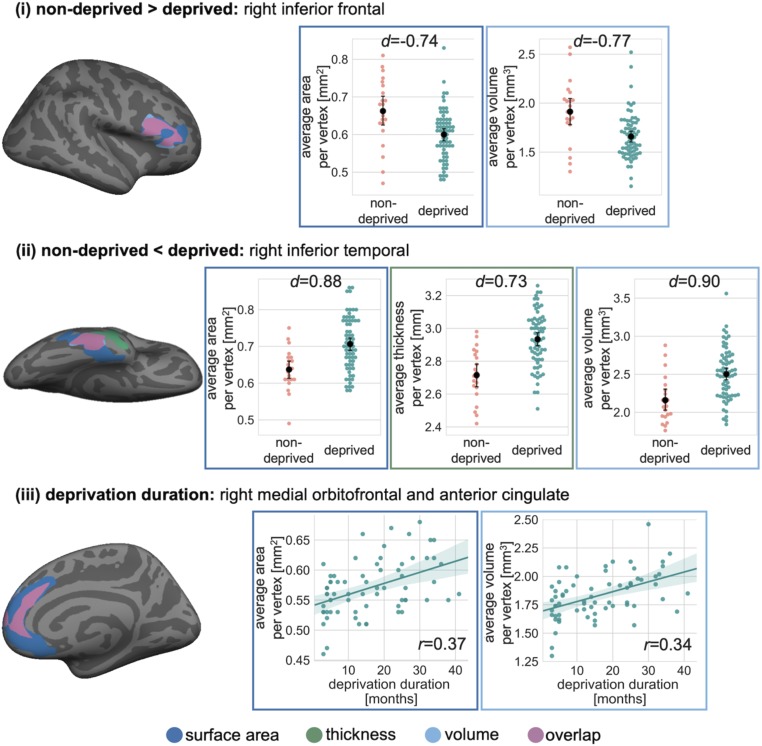
Deprivation-related regional differences in cortical volume, thickness, and surface area. (*Top*) Relative to nondeprived UK adoptees, the deprived Romanian adoptees had smaller surface area and volume in a cluster in the right inferior frontal gyrus. (*Middle*) The deprived Romanian adoptees had greater cortical thickness, surface area, and volume in a cluster in the right inferior temporal gyrus. (*Bottom*) There was a positive correlation between deprivation duration and cortical surface area and volume of the right medial prefrontal cortex. This cluster included the right superior frontal, medial orbitofrontal, and anterior cingulate cortices. Brain maps are displayed in *Left*. Point and swarm plots in *Right* display averages of vertexwise measures of each cluster, with dots representing individual participants (*n* = 88). Black whiskers show 95% CIs around the means (black dots). All clusters were significant on a whole-brain level following correction for multiple comparisons (clusterwise threshold *P* < 0.05). Effect sizes (Cohen’s *d* and Pearson’s *r*) of each cluster were derived from whole-brain vertexwise effect size brain maps. All analyses included TBV (except cortical thickness) and sex as covariates. Individual data points represent measures after regressing out these covariates.

Adopting a whole-brain surface-based morphometry (SBM) approach, we identified 2 additional deprivation-related regional alterations after including TBV as a covariate in the cortical volume, surface area, and gyrification analyses (as these, but not cortical thickness, scale closely with TBV) (32). Relative to the nondeprived UK adoptees, the institutionally deprived Romanian adoptees showed 1) significant reductions (over and above general TBV effects) in surface area and volume in the right inferior frontal gyrus extending into the middle rostral frontal gyrus and 2) significantly greater cortical surface area, thickness, and volume in a cluster extending from the right inferior temporal gyrus into the parahippocampus and temporal pole (Fig. 2 and Table 1). There were no significant group differences in local gyrification.

**Table 1.** Clusters showing significant differences between the groups in cortical volume, thickness, or surface area

Measure	Anatomical region	H	Cluster size, mm^2^	Peak Montreal Neurological Institute (MNI) coordinates, mm			Clusterwise *P*	Effect size
				*x*	*y*	*z*		
Nondeprived > deprived								
Volume	Inferior frontal	R	1,269	55	17	16	0.0004	−0.77*
Area	Rostral middle frontal	R	1,859	42	25	21	0.0068	−0.74*
Nondeprived < deprived								
Volume	Inferior temporal	R	800	52	−26	−28	0.0331	0.90*
Area	Inferior temporal	R	1,708	44	−17	−25	0.0134	0.88*
Thickness	Inferior temporal	R	1,178	58	−27	−29	0.0022	0.73*
Deprivation duration								
Volume	Superior frontal	R	1,252	10	63	12	0.0004	0.34^†^
Area	Superior frontal	R	2,721	14	46	0	0.0002	0.37^†^

Monte Carlo correction for multiple comparisons was applied (clusterwise threshold *P* < 0.05). Effect sizes (Cohen’s *d* and Pearson’s *r*) were taken from whole-brain vertexwise effect size brain maps. H, hemisphere; R, right.

* Cohen’s *d*.

^†^ Pearson’s *r* (correlation coefficient).

### Associations between Local Cortical Structure and Deprivation Duration.

Whole-brain SBM analyses within the institutionally deprived group showed a positive correlation between deprivation duration and surface area and volume in the right medial prefrontal cortex, which included the right medial orbitofrontal cortex and rostral anterior cingulate cortex (Fig. 2 and Table 1). Given that we covaried for TBV in these analyses, these effects represent relative sparing of these regions in the context of more general global reductions. For cortical thickness and local gyrification, no significant clusters were identified that showed an association with deprivation duration.

### Testing for Local Alterations in Subcortical Regions.

After covarying for TBV and sex, there was no association between institutional deprivation and the volume of the subcortical regions investigated, namely the amygdala, hippocampus, thalamus, nucleus accumbens, caudate, putamen, and pallidum (deprived vs. nondeprived: all *P*_*FDR*_ > 0.40; duration of deprivation effects: all *P*_*FDR*_ > 0.65) (*SI Appendix*, Fig. S3).

### Do Alterations in Brain Structure Statistically Mediate the Relationship between Deprivation and ADHD Symptoms, ASD Symptoms, or IQ?

To address our second research question, we investigated whether deprivation-related alterations in global or local brain structure mediated the relationship between deprivation and IQ, ADHD, or ASD symptoms in 3 separate analyses. Compared with the nondeprived group, the deprived group had significantly higher levels of ADHD symptoms [*B* = 2.87, *F*(1,78) = 7.48, *P* = 0.008] and lower IQ [*B* = −11.36, *F*(1,86) = 9.66, *P* = 0.003], but there was no significant difference in ASD symptoms [*B* = 0.93, *F*(1,75) = 1.54, *P* = 0.22] (descriptive statistics are in *SI Appendix*, Table S1).

In the 3 path models performed using bootstrapped SEs and bias-corrected confidence intervals (CIs; 5,000 bootstraps), TBV significantly mediated the relationship between institutionalization and IQ (*n* = 88, *B* = −5.51, SE = 2.34, 95% CI = [−11.49, −1.67], *R*^2^ = 0.20). The direct relationship between institutionalization and IQ was no longer significant when including TBV in the model (*B* = −5.85, SE = 4.68, 95% CI = [−14.79, 3.46]). TBV also mediated the association between institutionalization and ADHD symptoms (*n* = 80, *B* = 0.93, SE = 0.55, 95% CI = [0.03, 2.24], *R*^2^ = 0.12). However, the direct relationship between institutionalization and ADHD symptoms remained significant in this model (*B* = 1.94, SE = 0.95, 95% CI = [0.08, 3.81]). Thus institutionalization-related reductions in TBV were associated with both lower IQ and elevated ADHD symptoms. TBV did not significantly mediate the association between institutionalization and ASD symptoms (direct: 95% CI = [−1.29, 1.58]; indirect: 95% CI = [−0.23, 1.25]).

We next examined whether local structural alterations mediated the relationship between deprivation and IQ, ADHD, or ASD symptoms. To do so, we extracted average volumes of the 3 cortical regions that showed deprivation-related alterations (inferior temporal, inferior frontal, and medial prefrontal clusters) and examined residuals after regressing out TBV and sex. As differences in inferior frontal and temporal volumes were related to institutionalization per se, we investigated whether they mediated the effect of group status (deprived vs. nondeprived) on neurodevelopmental outcomes, whereas we examined whether medial prefrontal volume mediated the relationship between deprivation duration and neurodevelopmental outcomes within the deprived group. This involved running 9 separate path models.

There was a significant indirect effect of institutionalization on ADHD symptoms via inferior temporal gyrus volume (*n* = 80, *B* = −1.62, SE = 0.60, 95% CI = [−3.03, −0.65], *R*^2^ = 0.22). As expected based on prior analyses, the direct pathway from institutionalization to ADHD symptoms indicated that deprivation was associated with more ADHD symptoms (*B* = 4.48, SE = 0.93, 95% CI = [2.73, 6.43]). In contrast, the indirect pathway suggested that, where deprivation was associated with relative sparing of the inferior temporal gyrus (as also shown above), this was associated with lower levels of ADHD symptoms—suggesting that the deprivation-related alterations in that region may be compensatory in nature. Neither of the other regions investigated significantly mediated the relationship between institutionalization and ADHD symptoms or the relationship between deprivation duration and ADHD symptoms within the deprived group alone. None of the local cortical volumes significantly mediated the relationship between institutionalization or deprivation duration and IQ or ASD symptoms.

## Discussion

This study provides evidence that exposure to severe deprivation, which is limited to the first years of life, is associated with profound and enduring alterations in brain volume and structure in young adulthood. Such alterations were clearly detectable even when individuals exposed to this form of deprivation were subsequently brought up in families that provided nurturing environments for the rest of their childhoods. Not only did previously deprived adoptees have substantially smaller brains than their nondeprived counterparts, but the degree of reduction in TBV increased linearly with each additional month of deprivation. This association remained significant after covarying for a range of possible confounds.

Largely based on animal experimental models (33), a hypothesis has emerged that adverse environments experienced during sensitive periods in early childhood produce enduring effects on the brain (34), which increase the risk for psychopathology in the long term (11). Such time-limited effects could have a number of different causes. They could be due to the absence of experiences thought necessary for normal development (experience-expectant programming) or because of anticipatory adaptation of the brain to future adversity (experience-adaptive programming) (2). Alternatively, they could be due to subtle forms of damage (so-called “neural scars”), perhaps linked to the toxic effects of stress on the developing brain (4).

While our data suggest that the effects of exposure to early adversity may not be fully remediable by later environmental enrichment (2), they do not allow us to distinguish between these different explanations. Certainly, the institutions deprived children of formative experiences regarded as necessary for normal brain development (consistent with the experience-expectant programming hypothesis). However, individuals were also likely to have experienced chronic stress, which could have led to alterations in brain structure that may not be reversible. We were also unable to specifically test the sensitive period hypothesis (35), because the children all entered the institutions at around the same time, meaning that the impact of exposure during different developmental windows could not be compared.

There are a number of other possible explanations for our findings. First, observed TBV differences between deprived and nondeprived groups might result from ethnic differences in head size norms between Romanian and UK adoptees. Normative differences in head circumference between European countries have been observed (36). However, leaving aside the fact that such differences could not account for linear deprivation duration effects within the Romanian group, such ethnic differences are far too small to account for the large group-related deprivation effects on TBV (Cohen’s *d* = −1.13) seen here.

Second, the association between deprivation and brain volume might reflect a nonspecific delay in growth as seen in the effects of deprivation on height (37). The fact that the association between deprivation and TBV was not explained by variation in height suggests that this was not the case.

Third, deprivation exposure might be correlated with genetic or prenatal risk for smaller brains, and this rather than the deprivation exposure itself might have driven deprivation-related findings in TBV. Such an explanation is not consistent with the finding that the TBV effects were independent of birth weight (a proxy for intrauterine exposure) and polygenic scores for intracranial volume.

Fourth, it is possible that smaller brain volumes are caused by subnutrition within the institutions rather than by social deprivation. Certainly, a large proportion of the Romanian adoptees lacked sufficient food during their time in the institutions, as many were severely underweight when placed for adoption (14). However, there was no strong evidence that TBV reductions were linked to subnutrition defined in this way. Because we were unable to measure the composition of the individual adoptees’ diets, we could not test the impact of diet on early brain growth.

These supplementary analyses suggest that the causes of the global reductions in brain volume observed in the Romanian adoptees were largely psychosocial in nature rather than reflecting subnutrition, prenatal or genetic risk, or ethnic differences in brain size.

As predicted based on previous studies in nondeprived samples (28, 29), the relationship between institutional deprivation and both low IQ and ADHD symptoms was mediated by reductions in TBV. Explanations of both individual variations in and the known relationships between IQ, ADHD, ASD, and TBV have tended to focus on the role of genetic factors based on evidence from twin studies in normative samples showing that they are highly heritable traits (38–40). However, we know that heritability estimates vary considerably as a function of the characteristics of populations, with lower estimates seen in nonnormative populations exposed to unusual levels of environmental risk (41). This supports the notion that extraordinary environments have the potential to override underlying genetic liability—presumably through either epigenetic or brain programming effects (42). The most convincing evidence to date of such an environmentally driven effect linking TBV and development derives from studies of adolescents born extremely preterm who have smaller TBV and lower IQ, with TBV explaining about 30% of the difference in IQ between the preterm-born and control groups (43). Our results extend this account to highlight an equivalent role for social adversity as seen for prematurity and raise the possibility that 2 quite different environmental exposures produce similar effects on the brain, which drive low IQ (i.e., consistent with the concept of equifinality). It is worth noting that ADHD symptoms are also elevated in premature children (44). In a previous study, cortical thinning in frontal, parietal, and temporal regions was found to mediate the link between institutional deprivation and symptoms of inattention and impulsivity in children (23). This overall pattern of results seems in line with the link that we observed between TBV and ADHD symptoms in young adulthood, although we did not find evidence for cortical thinning in our sample. While we did not find significant links between deprivation or TBV and ASD, these findings should be interpreted cautiously, as they are likely due to statistical power limitations. Two points merit consideration here. First, this neuroimaging sample included only a subset of participants from the full ERA study sample. We did find an association between deprivation and adult ASD symptoms in the full sample (12). Second, the UK adoptees and the Romanian adoptees with limited exposure to deprivation both show very low levels of ASD symptoms.

After correcting for TBV, there were a number of localized deprivation-related alterations in brain regions of putative significance for neurodevelopmental and neuropsychological outcomes linked to adversity and maltreatment: relative increases in thickness, surface area, and volume of the right inferior temporal cortex (extending into the parahippocampal gyrus and temporal pole) and additional reductions in surface area and volume of the right inferior frontal cortex (extending into middle rostral frontal cortex). Moreover, longer deprivation duration was associated with relatively greater volume and surface area of the right medial prefrontal cortex. These regions have previously been shown to be affected by childhood maltreatment and adversity—although the nature/direction of the effects found here differed for 2 of 3 regions compared with previous results. Our finding of smaller right inferior frontal surface area and volume was consistent with prior findings of smaller dorsolateral and ventrolateral prefrontal cortex volumes in children, adolescents, and adults with a history of early maltreatment (45, 46). Studies implicating temporal cortex regions have also found reduced thickness and volume (46–48). This is contrary to our results showing greater thickness, surface area, and volume of the right inferior temporal cortex in the deprived group. Reductions in the volume of the anterior cingulate and medial prefrontal cortex have perhaps been most widely reported following early maltreatment (11, 47, 49–55)—while our findings of a positive association between deprivation duration and this region suggest that the volume and surface area of this region are relatively preserved following extended deprivation.

While, in general, these TBV adjusted regional variations were not related to IQ or ADHD symptoms, there was one striking exception. The relatively spared volume of the inferior temporal lobe in the institutionally deprived group was associated with lower levels of ADHD symptoms, with path analysis supporting a mediating role of structural changes in this region. This is consistent with the notion that some of the regional brain variations associated with institutional deprivation are the result of compensatory cortical restructuring occurring either within the institutions or in the adoptive homes and the broader hypothesis that some brain alterations observed in maltreated children are adaptive, as they often either occur in the absence of psychopathology or are actually associated with more positive outcomes (30). This study provides some evidence of such effects in humans. This highlights the double-edged nature of brain plasticity—while leaving individuals vulnerable to the effects of adversity (in this case, institutional deprivation), it also offers the promise of recuperation and recovery.

It was notable that we found no effects of deprivation on subcortical structures in our adult sample. Previous studies have highlighted the potential vulnerability of the limbic system to early maltreatment: Many report smaller hippocampal volume, but findings are inconsistent for amygdala volume (4, 11, 56). However, we found no evidence of associations between deprivation and amygdala or hippocampal volume. There are a number of possible explanations for the disparity between these findings and those from previous studies in children and adolescents. These include the nature (extreme neglect) and timing (very early in life) of the deprivation, possible genetic confounding between maltreatment exposure and brain-related risk in previous studies, the age at follow-up (with effects potentially changing and diminishing over time), and/or a failure to properly control for co-occurring psychopathology. Finally, it is possible that some of the previous studies in this area that have reported regional effects failed to adequately control for the global effects of deprivation on TBV.

Why are certain brain areas particularly sensitive to early institutional deprivation? One potential reason could be their particularly rapid development during the first 2 y of life. Surface area of the regions observed here (right anterior cingulate, medial orbitofrontal, inferior frontal, and inferior temporal cortices) increases rapidly compared with the rest of the cortex (25). However, other brain areas, such as superior parietal cortex, develop even more rapidly in the first 2 y of life and did not seem to be sensitive to early deprivation in this study. Hence, early rapid growth rates are unlikely to be the only factor indexing vulnerability to early life stress. In later development, cortical thickness starts to decrease from the age of 2 y onward as result of synaptic pruning, while surface area continues to increase until the age of 11 to 15 y before it starts to gradually decline (3). As the brain imaging assessments within this cohort were cross-sectional, it was not possible to identify whether relatively greater surface area and volume of inferior temporal and medial prefrontal cortices reflect enhanced growth in early childhood in the period following adoption or a reduction in the typical pruning and volume loss observed in late childhood and adolescent development (or a combination of both). Likewise, smaller surface area and volume of the right inferior frontal cortex might reflect reduced growth in early life or increased volume loss in later childhood.

This study had many strengths: its design overcomes many of the limitations of previous studies of maltreatment and early adversity and includes a nondeprived control group of UK adoptees, which allowed us to isolate the effect of early deprivation from later adverse experiences on the one hand and adoption per se on the other. The timing of placement into adoptive families was carefully recorded, which enabled us to test for deprivation duration effects within the Romanian adoptee group. We were also able to relate imaging data with clinical and IQ data obtained from the same individuals and explore how changes in volume were associated with changes in cortical thickness, surface area, and folding. Nevertheless, the study had several limitations, which should be noted. First, despite the positive features of this study design, its necessary lack of an experimental approach means that we cannot definitively claim a causal link between deprivation and brain structure. This is the case even for the most compelling evidence of a causal effect—the dose–response associations between deprivation duration and TBV and medial frontal cortical surface area and volume. Given that the vast majority of children entered the institutions in the first few weeks of life, this variable is almost completely determined by the time that they were adopted and left the institutions (rather than when they entered them). Although in general, the timing of adoption was determined by historical events, there may be factors associated with late adoption that increased risk of brain growth abnormalities. We found no evidence that late-adopted children were at an increased prenatal risk (with birth weight as a marker for intrauterine growth) or genetic risk for smaller intracranial volume (as indicated by polygenic scores), but we cannot exclude the possibility that children adopted later were at increased risk for smaller brains due to other factors that were not measured. Second, because of the reduced sample size compared with the full adult follow-up sample, we were only adequately powered to detect medium or large effects. However, the sample is still relatively large compared with prior neuroimaging studies investigating institutional deprivation (19, 21, 22). Third, although embedded within a prospective longitudinal design, the neuroimaging aspect of this study was cross-sectional. Longitudinal neuroimaging studies are needed to investigate how childhood deprivation impacts brain developmental trajectories and examine the stability of structural alterations observed following deprivation exposure. Fourth, it should be noted that we were only able to investigate ADHD and ASD on a symptom rather than a diagnosis level, and symptoms were assessed using parent-rated questionnaires rather than clinical interviews. By not assuming a clinical diagnosis as a categorical cutoff, we were able to investigate symptoms as a continuum, but we cannot make inferences regarding ADHD or ASD, as clinical disorders per se and comparability with studies using groups of clinically diagnosed patients might be limited.

In conclusion, we have shown that—more than 20 y after it ended and was replaced by environmental enrichment in adoptive families—institutional deprivation was associated with smaller TBV and regional cortical alterations in young adulthood. TBV alterations mediated the relationship between institutionalization and lower general intelligence and higher levels of ADHD symptoms in adulthood. The possibility that these associations could be caused by some set of confounding factors linked to early deprivation (not accounted for or controlled in this study) rather deprivation itself cannot be ruled out definitively. That said, these findings are consistent with the hypothesis that time-limited and severe adversity, experienced in the first years of life, can have an enduring adverse effect on brain development that is still observable in adulthood. The results also raise the possibility that regional compensatory effects may protect some institutionally reared children from developing ADHD.

## Materials and Methods

### Participants.

The original ERA sample included 165 Romanian adoptees (Rom) and a nondeprived control group of 52 UK adoptees (UK) placed for adoption before 6 mo. Of these, 81 Rom and 23 UK took part in the English and Romanian Adoptees Brain Imaging Study (ERABIS); 11 Rom who had never been institutionalized but were directly adopted from Romanian families were excluded from the analysis. Their brain volumes showed a significantly higher variance compared with the previously institutionalized Rom, indicating that their preadoptive environment might not be comparable. Moreover, 2 UK and 3 Rom were excluded from analysis due to missing structural MRI data. The final sample included 67 Rom (40.6% of the original sample, 50.7% female, mean age = 25.3 y, age range = 23 to 28 y) and 21 UK (40.4% of the original sample, 38.1% female, mean age = 24.4 y, age range = 23 to 26 y). Most Rom entered the institutions in the first few weeks of life. Deprivation duration was, therefore, estimated based on the age in months at which adoptees first entered a household in the United Kingdom. For the Rom group seen in the ERABIS, deprivation duration ranged between 3 and 41 mo.

Data collection took place at the Centre for Neuroimaging Sciences at King’s College Hospital, London. All participants gave written informed consent to participate and received a £100 Amazon voucher as reimbursement for their time. The ERABIS received ethical approval from the ethics committee of the University of Southampton and the Camberwell–St. Giles National Health Service Research Ethics Committee (ethics no. 14/LO/0477).

### Measures.

#### Physical growth.

Height (in centimeters) was recorded in young adulthood during the latest ERA follow-up study, the ERA young adult follow-up when participants were aged between 22 and 26 y. Birth weight (in kilograms) was obtained from Romanian reports (57).

#### Subnutrition.

Weight was recorded when children entered the United Kingdom soon after leaving their institution and measured as SD from age- and sex-adjusted UK norms (58). At that time, ∼69% of Rom suffered from subnutrition, with weight at more than 1.5 SDs below UK norms.

#### Polygenic scores for intracranial volume.

DNA samples were obtained with self-collection buccal cell kits and genotyped with the Illumina Infinium PsychArray-24 Kit. Polygenic scores for intracranial volume were calculated with PRSice (59) and based on summary statistics from the Enhancing NeuroImaging Genetics Through Meta-Analysis (ENIGMA) genome-wide association study (60). Individual scores represent sum scores of the intracranial volume-associated effect sizes of the single-nucleotide polymorphisms (SNPs). The optimal (explaining most of the phenotypic variance) probability threshold for inclusion of SNPs was based on the TBV data available in this sample.

#### Deprivation-specific neurodevelopmental problems.

Young adult symptoms of ADHD, ASD, and DSE are significantly associated with deprivation (12). Cognitive impairment was associated with deprivation earlier in development but had remitted considerably by young adulthood (12). *SI Appendix*, Table S1 has an overview of data available, and *SI Appendix*, Table S2 has a list of all items used per symptom domain.

ADHD symptoms were measured with the 20 parent-rated items of the Conners Comprehensive Behavior Rating Scales (CBRS; 0 to 18 scale) (16, 61). Items reflect the 18 Diagnostic and Statistical Manual of Mental Disorders (DSM-5)5 ADHD symptoms and were adapted for young adults with permission from the copyright holders (16).

ASD symptoms were assessed with 15 items of the parent-rated Social Communication Questionnaire (SCQ), which had previously been judged as developmentally relevant for young adults (0 to 15 scale) (12, 62).

DSE symptoms were rated based on parents’ responses to 3 interview questions, which explored the construct of being “too friendly towards strangers,” “inappropriately intrusive,” and “unaware of social boundaries,” Responses to each question were rated as endorsed (1) or not endorsed (0; 0 to 3 scale) (12).

IQ was assessed as a measure of cognitive impairment with the Wechsler Abbreviated Scale of Intelligence, Second Edition (63), which is a widely used and reliable test of general intelligence.

### Procedure.

Participants were recruited via mail and phone, and they and their families were invited to come to London to take part in this study. The ERABIS protocol involved 2 MRI scanning sessions, which took approximately 1 h each, and were typically done on consecutive days. In most cases, the structural scan was acquired at the beginning of the first scanning session after participants were familiarized to the scanning environment. There was also a neuropsychological testing and questionnaire session, which took ∼6 h and included an assessment of IQ. Adoptive parents filled in the Conners CBRS and the SCQ and answered the DSE interview questions during the previous follow-up study, the ERA young adult follow-up.

### MRI Data Acquisition and Processing.

Structural images were acquired on a General Electric MR750 3.0-Tesla MR scanner with a 12-channel head coil. We acquired 1 T1-weighted 3-dimensional Magnetization Prepared-Rapid Gradient Echo scan per participant (scanning parameters: repetition time (TR)/echo time (TE) 7,312/3.02 ms, flip angle 11°, 256 × 256 matrix, 1.2-mm thick, 196 sagittal slices, field of view = 270). Cortical thickness, surface area, volume, and local gyrification index as well as subcortical volumes were quantified using FreeSurfer 6.0.0 (http://surfer.nmr.mgh.harvard.edu). The procedure has been described in detail elsewhere (64–68). Smoothing was performed with a 10-mm kernel at full width/half max (FWHM) for cortical thickness, surface area, and volume. As local gyrification index already is a smooth measure, we only applied a 5-mm FWHM kernel for smoothing.

### Statistical Analysis.

#### Global and regional alterations following institutional deprivation.

##### TBV and total gray and white matter volumes.

Analyses were performed in R 3.5.0 (69). To test the first set of hypotheses, general linear models were used to test for differences between deprived (Rom) and nondeprived (UK) groups in TBV and total gray and white matter volumes. Furthermore, linear regressions were performed within the deprived group to test whether these measures correlated with deprivation duration when used as a continuous measure. Next, body height was added as covariate in a general linear model to test whether deprivation duration was related to TBV after controlling for these factors. In subsequent analyses, we tested whether deprivation duration was associated with birth weight or polygenic scores for intracranial volume. We also tested whether TBV was predicted by subnutrition (weight at UK entry). We then tested whether deprivation duration predicted TBV if additionally adding birth weight, polygenic scores for intracranial volume, or weight at UK entry as covariates to the model. Sex was entered as a covariate in all analyses, as findings of sex differences in brain volume are well established (70).

##### Regional cortical alterations.

To test for regional cortical alterations following institutional deprivation beyond global effects, in a whole-brain surface-based approach, we first tested for differences between deprived and nondeprived groups in cortical thickness, surface area, volume, and local gyrification using general linear models. Also, whole-brain linear regression analyses were performed within the deprived group to investigate if there was a linear relationship between duration of deprivation and any of these measures. These analyses were performed with FreeSurfer 6.0.0.

In addition to sex, TBV was entered as a covariate for volume, surface area, and local gyrification measures (as these, but not cortical thickness, are linearly related to TBV) (32) to examine whether there were regional differences between the groups that were not proportional to global brain volume. For all whole-brain analyses, clusterwise correction for multiple comparisons was performed using a Monte Carlo simulation (vertexwise threshold *P* < 0.05, clusterwise threshold *P* < 0.05).

##### Subcortical volumes.

We tested for differences between the deprived and nondeprived groups in relative subcortical volumes (including sex and TBV as covariates) in general linear models. The volumes examined were the amygdala, hippocampus, thalamus, nucleus accumbens, caudate nucleus, putamen, and pallidum for the left and right hemispheres separately. Furthermore, partial correlations were performed to identify if deprivation duration was related to relative subcortical volumes (covarying for sex and TBV). Each set of models was corrected for multiple comparisons using the false discovery rate procedure (q = 0.05) (71).

#### Relationship to neurodevelopmental outcomes.

##### TBV.

To answer our second research question, we next investigated whether TBV mediated deprivation-specific neurodevelopmental outcomes. In this study, we investigated symptoms of ADHD, ASD, and IQ. Symptoms of DSE have also been related to institutional deprivation. However, as there was only 1 case with DSE symptoms in the UK adoptees group, it was not possible to perform mediational analyses. We first tested for group differences (deprived and nondeprived) in IQ or symptoms of ADHD or ASD using 3 general linear models. We then used path model analysis in a structural equation model framework to investigate whether TBV statistically mediated the relationship between institutionalization and IQ, ADHD, or ASD symptoms (in 3 separate models). Using the “lavaan” package (72), 5,000 bootstraps were performed to compute (bias-corrected) CIs and SEs for indirect and direct effects. The influence of sex on TBV was removed before entering the residuals into the model. As all mediation models were just identified, no model fit indices were computed.

##### Regional cortical alterations.

All significant clusters identified as sensitive to institutionalization or deprivation duration were selected as regions of interest, and the averages of vertexwise volumes of each cluster were extracted for every participant regressing out the covariates TBV and sex. We ran path models as above to test if regions of interest mediated the relationship between institutionalization or deprivation duration and IQ or ADHD or ASD symptoms (using 3 separate models per region of interest, resulting in 9 models in total).

### Data Availability.

The data that support the findings of this study are deposited with King’s College London’s Research Data Management System (https://doi.org/10.18742/RDM01-497) and will be shared by email request.

## Supplementary Material

Supplementary File
