# Supplementary material for: Early childhood deprivation is associated with alterations in adult brain structure despite subsequent environmental enrichment
Source: Proc Natl Acad Sci U S A. 2020 Jan 6;117(1):641–9. doi: 10.1073/pnas.1911264116 (PMC6955353; doi:10.1073/pnas.1911264116)
Supplement: Supplementary File [file pnas.1911264116.sapp.pdf]

PNAS
www.pnas.org

Supplementary Information for

Early childhood deprivation is associated with alterations in adult brain structure despite subsequent environmental enrichment

Nuria K. Mackes, Dennis Golm, Sagari Sarkar, Robert Kumsta, Michael Rutter, Graeme Fairchild, Mitul A. Mehta, Edmund J. S. Sonuga-Barke, on behalf of the ERA Young Adult Follow-up team

Correspondence should be addressed to Edmund Sonuga-Barke or Nuria Mackes, Department of Child and Adolescent Psychiatry, Institute of Psychiatry, Psychology and Neuroscience, King's College London, 16 De Crespigny Park, SE5 8AF, London, UK, E-mail: edmund.sonuga-barke@kcl.ac.uk, nuria.mackes@kcl.ac.uk, phone: +44 203 228 0464.

This PDF file includes:

Supplementary methods
Figures S1 to S3
Tables S1 to S2
The ERA Young Adult Follow-up team
SI References

www.pnas.org/cgi/doi/10.1073/pnas.1911264117

Supplementary methods: MRI acquisition and processing

Initial quality control. All structural MRI scans were visually inspected. During acquisition, we initially assessed whether the scan needed to be repeated due to excessive head movement. After the assessment, for each scan, we recorded (a) whether the whole brain had been scanned, (b) motion artefacts, (c) hypo- and hyper-intensities, (d) ghosting (signal outside of the brain) and (e) other artefacts. If two or more structural T1 scans were available, the higher quality one (usually the last) was chosen for further analysis. All participants who completed MRI scanning had structural T1 scans suitable for further processing.

Quality control and editing. After automated surface- and volume-based segmentation with FreeSurfer 6.0, a second quality control step was performed using a QA tool script, which we updated for FreeSurfer 6.0 (https://surfer.nmr.mgh.harvard.edu/fswiki/QATools). Even though FreeSurfer is a highly robust software package, there can be failures in segmentations. Common failures include: skull strip errors (either brain instead of skull was removed or not enough skull was removed), white matter surface segmentation errors, intensity normalisation errors and pial surface misplacement. The quality of each segmentation was assessed by first confirming that all processing steps had been completed and all files created. If there were missing files, the required processing steps were repeated. Then, all segmentations were visually assessed using the snapshot tool provided as part of the QA tool. Segmentations that showed failures such as the ones described above were marked for editing. Afterwards, potential segmentation failures were determined by calculating outliers (more than 2 SD from the mean) of the main cortical and subcortical volumes. The signal-to-noise ratio for white matter registration and average white matter intensity was also calculated. Segmentations with outliers, low signal-to-noise-ratios or deviating white matter intensities were inspected more closely for failures. Overall, we aimed to edit as little as possible to keep reliability of segmentations high. It has been shown that editing has only little impact on the resulting summary measures and might introduce bias in itself (1). Fortunately, segmentation quality was high throughout and required only minimal editing: For four scans, voxels were manually added to the brain mask as too much had been removed as part of the skull stripping process. For 21 scans, the white matter surface was extended to comprise erroneously excluded white matter due to failed intensity normalisation mostly by changing intensity values of voxels. The researcher performing quality control and editing was blind to group status.

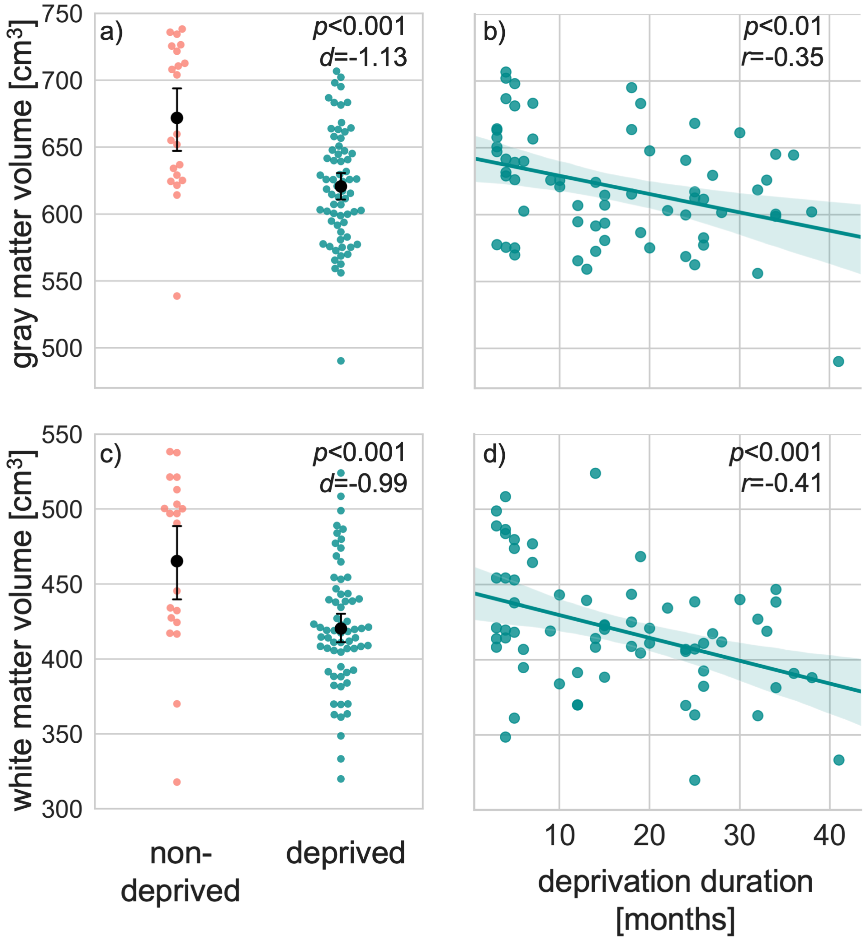

Fig. S1: Deprivation-related differences in gray and white matter volumes. Point- and swarm-plot depicting the distributions of gray matter volume (a) and white matter volume (c) in the non-deprived UK and deprived Romanian adoptees groups (gray: $F(1,85)$=20.43, p<0.001; white: $F(1,85)$=15.69, p<0.001). Black whiskers show 95% confidence intervals around the means (black dots). Negative correlation between deprivation duration and gray matter volume (b), and white matter volume (d) (gray: β=-0.26, $r_{partial}$=-0.35, $t(64)$=-3.00, p=0.004, white: β=-0.35, $r_{partial}$=-0.41, $t(64)$=-3.58, p=0.001). The shaded area depicts the 95% confidence interval around the regression line. The effect of sex has been regressed out for all volumes. Effect sizes were calculated with Cohen's d and Pearson's r.

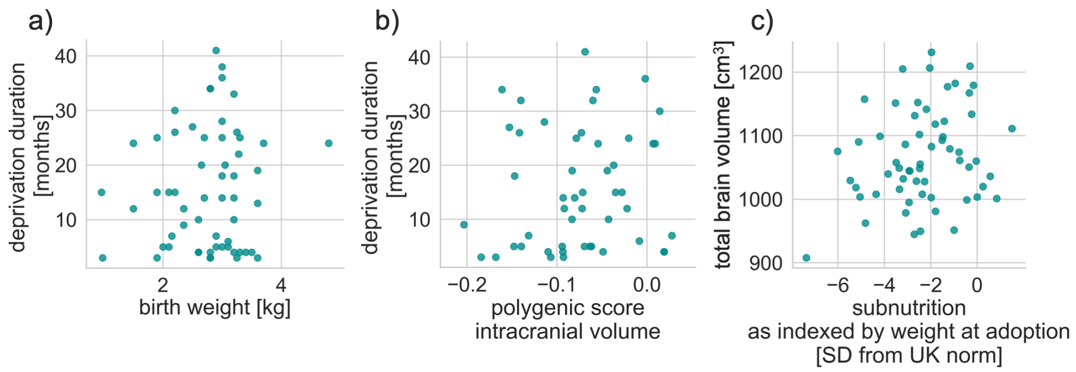

Fig. S2: Possible influences for smaller total brain volume and deprivation duration. Neither birth weight (a, *N*=58) nor polygenic scores for intracranial volume (b, *N*=49) were significantly related to how long adoptees spent in the institutions. c) Subnutrition during institutionalization did not predict adult TBV (*N*=60). The effect of sex has been regressed out for TBV.

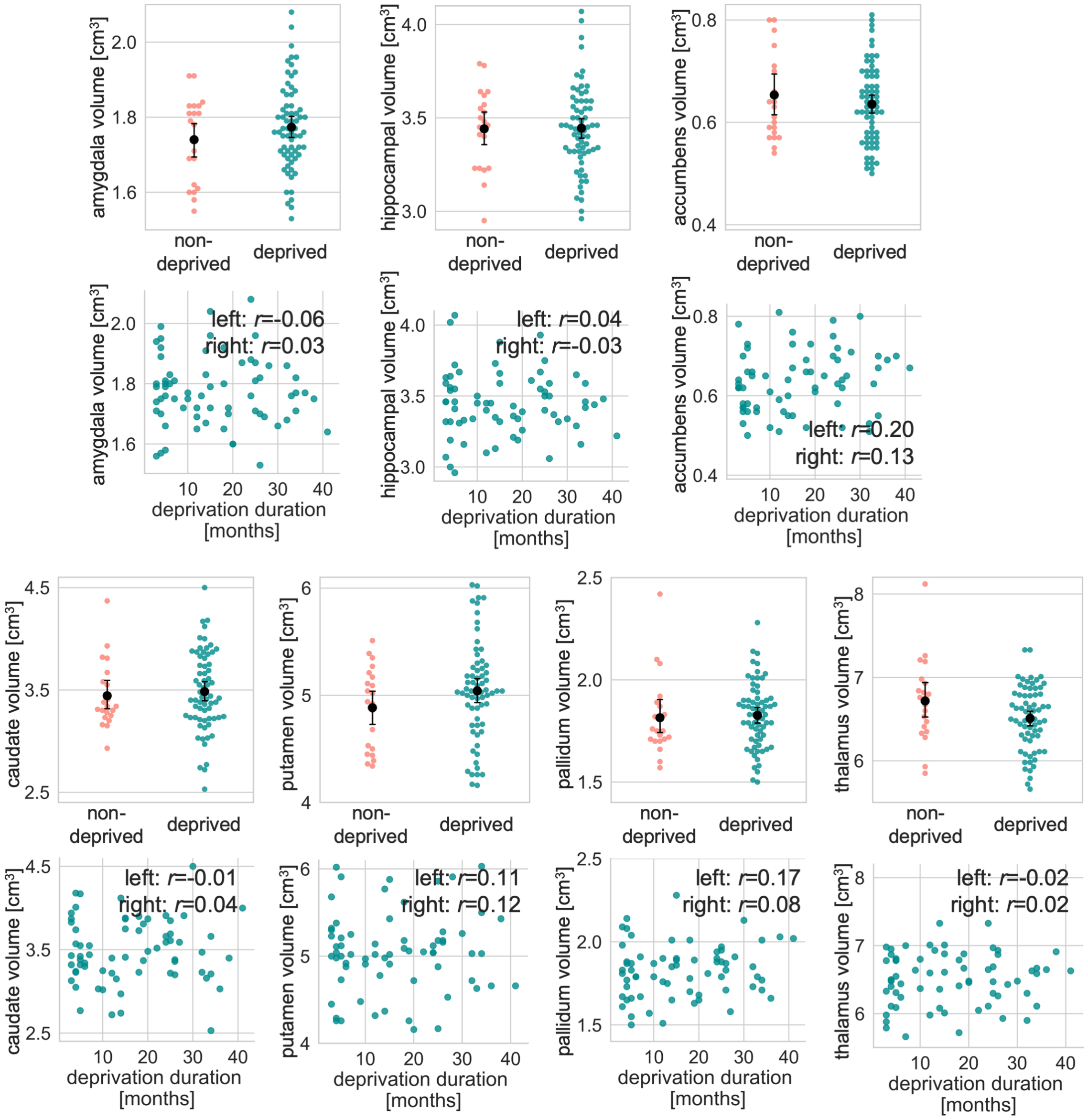

Fig. S3: Subcortical volumes. For illustration purposes, the average volume of left and right structures for each individual is displayed while analyses were performed for each structure and hemisphere separately. From left to right, up to down, the subcortical volumes are amygdala, hippocampus, nucleus accumbens, caudate, putamen, pallidum, and thalamus. Upper panel of each volume: there were no significant group differences between non-deprived UK adoptees and deprived Romanian adoptees in subcortical volumes. Whiskers represent 95% confidence intervals around the mean (black dot). Lower panel of each volume: Deprivation duration was not correlated with subcortical volumes. All analyses controlled for TBV and sex and individual data points represent volumes after regressing these covariates.

Table S1: Descriptive statistics for non-deprived UK and deprived Romanian adoptees groups. Significant group differences are marked in bold.

measure	group	n	mean	sd	median	range	group difference
deprivation duration	non-deprived			NA			NA
	deprived	67	16.19	10.98	15.00	38.00	
birth weight [kg]	non-deprived			NA			NA
	deprived	58	2.76	0.68	2.90	3.84	
weight at UK entry [SD from UK norms]	non-deprived			NA			NA
	deprived	60	-2.32	1.81	-2.41	8.86	
young adult body height [cm]	non-deprived	21	177.55	8.99	177.50	34.70	**$F(1,75)$=45.20, p<0.001**
	deprived	57	164.09	9.25	163.83	45.50	
young adult ADHD symptoms	non-deprived	20	1.20	2.31	0.00	8.00	**$F(1,78)$=7.48, p=0.008**
	deprived	60	4.07	4.48	2.00	15.00	
young adult ASD symptoms	non-deprived	20	1.05	3.25	0.00	14.00	$F(1,75)$=1.54, p=0.22
	deprived	57	1.98	2.76	1.00	12.00	
young adult DSE symptoms	non-deprived	19	0.11	0.46	0.00	2.00	NA
	deprived	59	0.51	0.92	0.00	3.00	
young adult IQ	non-deprived	21	106.71	15.96	112.00	77.00	**$F(1,86)$=9.66, p=0.003**
	deprived	67	95.36	14.17	96.00	72.00	

n – sample size with data available; sd – standard deviation; NA – not available

Table S2: Items used for each symptom domain at young adulthood. 18 DSM-5 ADHD symptoms were measured with 20 parent-rated items of the Conners Comprehensive Behaviour Rating Scales. ASD symptoms were assessed with 15 items of the parent-rated Social Communication Questionnaire. DSE symptoms were assessed with 3 interview questions.

**Attention-deficit/hyperactivity disorder symptoms**	
Inattention	*Hyperactivity*
1. Forgetfulness (1 item)	1. Cannot wait to answer (1 item)
2. Makes careless mistakes (1 item)	2. Cannot stay seated (1 item)
3. Lack of organization (1 item)	3. Restlessness (1 item)
4. Avoidance of tasks that require sustained effort (1 item)	4. Cannot wait for their turn (1 item)<br>5. Talking too much (1 item)
5. Unable to listen (1 item)	6. Fidgety (1 item)
6. Loosing things (1 item)	7. Noisiness (1 item)
7. Cannot sustain attention (1 item)	8. Interruption (1 item)
8. Easily distracted (1 item)	9. Constant movement (at least one of two items)
9. Unable to complete tasks (two items)	

**Autism spectrum disorder symptoms**		
Communication	*Social Reciprocal Interaction*	*Repetitive and Stereotyped Behaviors*
9. Odd speech<br>10. To and fro conversation<br>11. Socially appropriate<br>12. Difficulties with pronouns<br>13. Uses made up words/ phrases	17. Smiles back<br>21. Attempts to comfort<br>23. Normal range of facial expressions<br>24. Appropriate facial expressions<br>28. Responds positively to others	33. Odd interests<br>34. Interested in parts of objects<br>35. Ritualized behavior<br>36. Unusual interest in smell etc. of things or people<br>38. Odd mannerisms/ movement

**Disinhibited social engagement symptoms**
1. Seemed to friendly with strangers or too eager to approach strangers?
2. Made very personal comments or asked intrusive questions of others they've just met?
3. Seemed aware of social boundaries or the closeness of interaction with whom they are not familiar?

The ERA Young Adult Follow-up team

The ERA Young Adult Follow-up team is Edmund Sonuga-Barke, Mark Kennedy, Jana Kreppner, Nicky Knights, Robert Kumsta, Barbara Maughan, and Wolff Schlotz.

References

1. C. S. McCarthy *et al.*, A comparison of FreeSurfer-generated data with and without manual intervention. *Frontiers in Neuroscience* **9** (2015).
